# Evaluation of ColdZyme® Mouth Spray on prevention of upper respiratory tract infections in a boy with primary immunodeficiency: a case report

**DOI:** 10.1186/s13256-016-1085-2

**Published:** 2016-10-31

**Authors:** Mats Clarsund, Ulf Blom, Ann Gardulf

**Affiliations:** 1 Enzymatica AB, Ideon Science Park, 223 70 Lund, Sweden; 2 The Unit for Clinical Nursing Research and Clinical Research in Immunotherapy, Division of Clinical Immunology, Department of Laboratory Medicine, Karolinska Institutet at Karolinska University Hospital, Huddinge, Stockholm, 141 86 Stockholm Sweden

**Keywords:** Subcutaneous immunoglobulin therapy, Common variable immunodeficiency, Respiratory tract infections, ColdZyme® Mouth Spray, Quality of life

## Abstract

**Background:**

Primary immunodeficiencies include a variety of disorders that render patients more susceptible to infections. If left untreated, these infections may be fatal. Patients with primary antibody deficiencies are therefore given prophylactic immunoglobulin G replacement therapy. ColdZyme® Mouth Spray is a medical device intended to reduce the probability of catching a cold and/or can help shorten the duration of a cold, if used at an early stage of the infection, by forming a thin protective barrier on the pharyngeal mucous membrane. This is the first report of this kind in the literature.

**Case presentation:**

The parents of a 12-year-old white boy diagnosed as having common variable immunodeficiency voluntarily started to let their son use ColdZyme® Mouth Spray to reduce common cold infections if possible. Prior to using ColdZyme® Mouth Spray, he had recurrent microbial infections of his ears, sinuses, nose, bronchi, and lungs. He also frequently exhibited continuous rhinorrhea, fungal growth in his oral cavity, and gingivitis with wounds in his gums. As a consequence, his and his family’s health-related quality of life was severely compromised. He commenced a twice-daily treatment (morning and evening) with ColdZyme® Mouth Spray; the weekly administration of immunoglobulin G (Hizentra®) for replacement therapy was continued throughout this period. Data were retrieved by using a daily diary about infections and symptoms. His guardians had recorded infection symptoms since he was diagnosed as having common variable immunodeficiency 10 years earlier to follow the effect of the immunoglobulin G treatment. Shortly after commencement of ColdZyme® Mouth Spray treatment, he experienced a marked improvement in symptoms and health-related quality of life. His continuous rhinorrhea disappeared, breathing through his nose was easier, oral fungal infection decreased, and wounds in his gum tissue healed for the first time in several years.

**Conclusions:**

We observed that when ColdZyme® Mouth Spray was used to reduce common cold viral infection in a patient with common variable immunodeficiency on immunoglobulin G replacement therapy, secondary microbial and fungal infections in his oral cavity and oropharynx were also reduced. A controlled study is warranted to confirm the observed results.

## Background

Primary immunodeficiency diseases (PIDDs) are diseases in which the immune system has a defect that is not secondary to infection, drug effects, toxin exposure, or another disease. Patients with common variable immunodeficiency (CVID) [1] are prone to upper and lower respiratory tract infections [2]. In infection-prone children the disease is defined by a serum (S) immunoglobulin (Ig) G level from 3 g/L to the lowest normal reference level for the laboratory and a S-IgA level between 0.07 g/L and the lowest normal reference level for the laboratory [3].

The Swedish National Board of Health and Welfare [4] estimated that there are approximately 200 to 300 individuals with CVID in Sweden; the Swedish Medical PID Society estimated that there are approximately 450 individuals with CVID in Sweden [4]. Children and adults with CVID require lifelong replacement treatment with IgG retrieved from donor blood [5]. In many countries, the IgG replacement treatment is given to patients, including children, as subcutaneous IgG (SCIG) infusions [5, 6]. However, in spite of an adequate IgG replacement therapy, administered as SCIG or intravenously, a subgroup of patients continues to have frequent respiratory tract infections. As an example, a 2011 study found that patients with PIDD treated with IgG (Hizentra®) [7] had an average of five infections a year [8]. There remains, therefore, a great need to prevent and/or alleviate upper respiratory infections in patients with PIDD.

In the upper respiratory tract, mucous membranes covered with secreted mucous provide an innate barrier to infection. Reinforcing this barrier may be a favorable treatment strategy. In this case report, we report the effect of creating a physical barrier on the pharyngeal mucosal membrane to prevent or reduce infections in the upper respiratory tract. The barrier was applied by using a mouth spray to deliver the barrier solution. The barrier consists of glycerol, buffer salts, and a psychrophilic cod trypsin, currently marketed as a medical device, ColdZyme® Mouth Spray (ColdZyme), by Enzymatica AB, Lund, Sweden. In the device’s formulation, the combination of glycerol and cod trypsin exerts a barrier function when applied to the pharyngeal mucous membrane [9]. Glycerol, a natural humectant, attracts and retains nearby liquid and virus particles via absorption due to its high osmotic activity, while trypsin partly degrades protein structures presented on the viral capsid, and thereby reduces the interaction potential between viral capsids and epithelial cells, resulting in a decreased viral load. The viral load is of importance as there is a dose–response function between exposed virus dose (viral load) and probability of getting infected [10]. A double-blind placebo-controlled study found that ColdZyme reduced rhinovirus infection in the pharynx by reducing the viral load in comparison to a placebo, and the number of days with common cold symptoms was reduced from 6.5 to 3 days in comparison to a placebo [9].

## Case presentation

A 12-year-old white boy diagnosed as having CVID voluntarily started to use ColdZyme 3 years ago in the hope that it would prevent infection by the common cold virus. Since the age of 2 he had received weekly SCIG infusions: Hizentra®, 100 to 150 mg/kg weekly. Five months before the treatment with ColdZyme, analysis showed his S-IgG level to be 6.70 g/L; 3 months before the treatment with ColdZyme his S-IgG level was 6.02 g/L. Prior to treatment with ColdZyme he had recurrent microbial infections of his ears, sinuses, nose, bronchi, and lungs. He frequently exhibited continuous rhinorrhea, fungal growth in his oral cavity, and gingivitis with wounds in his gums. As a consequence, his and his family’s health-related quality of life (HRQL) had been severely compromised and he usually needed to stay at home from school at least 1 day per week. The month of November was often a particularly challenging month for him because recurrent upper respiratory tract infections often developed into pneumonia. Prophylactic treatment with amoxicillin for 9 months had little effect on the recurrent infections.

His parents commenced a twice daily treatment (morning and evening) of him with ColdZyme. The dose of the mouth spray was lower than the recommended dose of six times per day according to the instructions on the label. Further, the 15 months of preventative use was outside the intended use range of ColdZyme; that is, not to be used for more than 30 consecutive days. Weekly symptoms (malaise, fever, earache, sore throat, rhinorrhea, gastrointestinal symptoms, dry cough, mucus cough, cold sores) were recorded using a two-graded scale (yes/no) in an infection diary. His guardians had recorded infection symptoms since he was diagnosed as having CVID 10 years earlier, to follow the effect of the IgG treatment. Thus, historical data on self-reported infection frequency were available. In addition, a HRQL-related status such as days spent at home from school was also recorded. His IgG replacement treatment continued on a weekly basis and after 27 weeks of ColdZyme treatment, measurement of his S-IgG showed a level of 7.63 g/L.

**Fig. 1:**
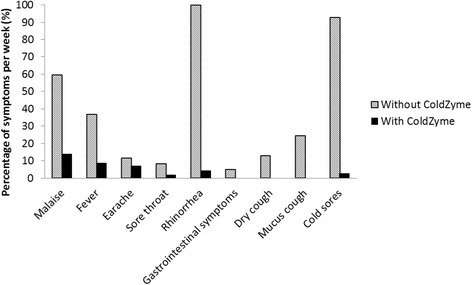
Infection symptoms per week. The average percentage of various infection symptoms per week for a 12-year-old boy diagnosed as having common variable immunodeficiency and treated weekly with subcutaneous immunoglobulin G (100 to 150 mg/kg/week). ColdZyme was used twice daily during a period of 15 months and the results are compared to historically collected data for a 21-month period prior to the ColdZyme treatment

Figure 1 shows the percentage of various infection symptoms he experienced per week during a period of 21 months prior to ColdZyme treatment and for the following 15 months when using ColdZyme. As shown, there was a pronounced reduction in self-reported infection symptoms during the ColdZyme treatment period; in particular, the percentage of symptoms of malaise, rhinorrhea, and cold sores. It was also noted in the infection diary that oral fungal infection decreased and wounds in his gum tissue decreased and healed. No adverse event was reported during the treatment period.

**Fig. 2:**
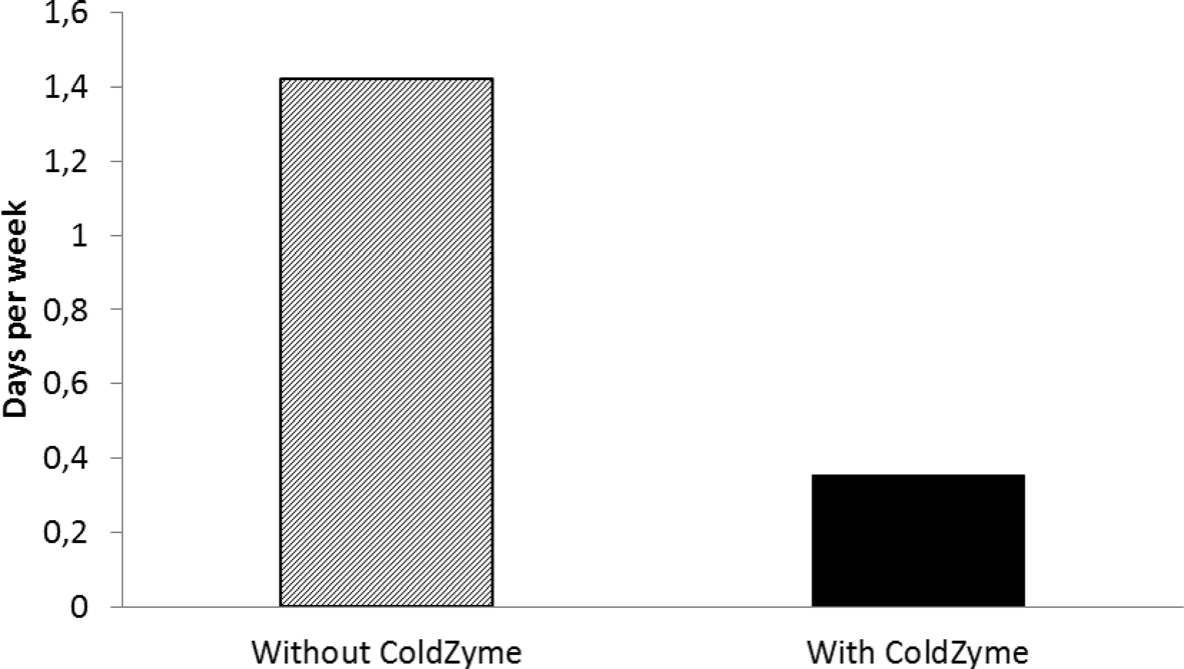
Absence from school. Average days per week spent at home from school for a 12-year-old boy diagnosed as having common variable immunodeficiency and treated weekly with subcutaneous immunoglobulin G (100 to 150 mg/kg/week). ColdZyme was used twice daily during a period of 15 months and the results are compared to historically collected data for a 21-month period prior to the ColdZyme treatment

Figure 2 shows the average number of days per week he was absent from school due to the severity of infection symptoms for the same periods as in Fig. 1. During the ColdZyme treatment period, he was on average away 0.3 days/week due to infections, as compared to an average of 1.4 days/week when not using ColdZyme.

## Discussion

This case report emphasizes the possible importance of the interplay between viral and bacterial species in infections and by targeting the viral infection it might also be possible to prevent secondary bacterial infections.

When a 12-year-old boy diagnosed as having CVID used ColdZyme, a medical device against the common cold, to prevent a common cold it was observed that it also had an impact on several infection symptoms. The effect was possibly obtained by strengthening his body’s natural innate barrier in his oral cavity and oropharynx toward infection. During the treatment period with ColdZyme he experienced a marked reduction in infection symptoms and an improved HRQL. His continuous rhinorrhea almost disappeared, breathing through his nose was easier, oral fungal infection decreased, and wounds in his gum tissue healed for the first time in several years. Such a quick onset of action and near total eradication of infection symptoms in our patient by treatment with ColdZyme was wholly unexpected, particularly given that the initial treatment period (October to November) coincided with the time of year during which he typically experienced the most severe infections. Understandably, he and his family were elated at the improvement in their HRQL following more than 10 years of debilitating recurrent infections. His IgG replacement therapy continued during the treatment with ColdZyme. His S-IgG level was found to be higher after nearly 7 months of treatment with the spray. One explanation of his higher S-IgG level might be that the reduction in frequencies of infections led to a decreased consumption of IgG and, therefore, a higher level of these antibodies in his blood circulation. It should be noted that his parents collected the historical data in the infection dairy before their son used ColdZyme without knowing that these data would be used to compare effects observed when using ColdZyme. He continues to use ColdZyme.

This is a single case study without a placebo control group and therefore the results are preliminary. Randomized double-blind placebo-controlled studies are warranted to confirm the observed results. This case report does not support a recommendation for using ColdZyme for treatment of primary immunodeficiency.

## Conclusions

Patients with CVID are prone to upper and lower respiratory tract infections. Respiratory infectious diseases are mainly caused by viruses or bacteria that often interact with one another. By using ColdZyme® Mouth Spray to reduce common cold viral infection it seems it is also possible to influence secondary microbial and fungal infections in the oral cavity and oropharynx.

### Perspective of the patient’s mother

The subcutaneous gamma-globulin treatment has been a great help for my son, but no matter how he was treated there were still some small symptoms left such as wounds in his oral mucosa and gums, which made him eat less and a constant runny nose that was not only annoying for him but which drove the whole family crazy.

Three years ago, my son began using ColdZyme on a daily basis and my intention was to see if this could help his immune system in fighting infections. This is exactly what happened and the number of mild infections declined.

What I did not count on was the unexpected positive add-on effects upon using ColdZyme. His mouth ulcers drastically decreased which gave me a very different son. He can now eat the same food as the rest of us almost every day without having pain and his weight and height has increased. Shortly after beginning treatment the constant runny nose symptom also disappeared.

Today, my son is a tall lanky teenager with hope in his eyes and lots of energy. He will probably always need his gamma-globulin treatment and I can see that ColdZyme helps him to reach the last, but very important, piece of well-being.

## Abbreviations

CVID: Common variable immunodeficiency
HRQL: Health-related quality of life
Ig: Immunoglobulin
PIDD: Primary immunodeficiency disease
S: Serum
SCIG: Subcutaneous immunoglobulin G
